# Relationship between the systemic immune-inflammatory index and overactive bladder risk: A cross-sectional assessment involving United States Adults

**DOI:** 10.1371/journal.pone.0323052

**Published:** 2025-05-07

**Authors:** Peng Zheng, Xiaoqian Wang, Junjie Ni

**Affiliations:** 1 Department of Vascular surgery, Affiliated Jinhua Hospital, Zhejiang University School of Medicine, Jinhua, Zhejiang, China; 2 Department of Endocrinology, Affiliated Jinhua Hospital, Zhejiang University School of Medicine, Jinhua, Zhejiang, China; 3 Department of Breast and Thyroid Surgery, Affiliated Jinhua Hospital, Zhejiang University School of Medicine, Jinhua, Zhejiang Province, China; China Medical University, TAIWAN

## Abstract

**Aims:**

This study aimed to evaluate the association between the systemic immune-inflammatory index (SII) and the risk of overactive bladder (OAB) in the adult United States population.

**Methods:**

Data from the National Health and Nutrition Examination Survey (NHANES) 2005–2010 were analyzed. A non-pregnant cohort aged ≥20 years with available SII and OAB data was included. Weighted univariate and multivariate logistic regression analyses were performed to assess the association between SII and OAB risk. Additionally, subgroup, interaction, and restricted cubic spline analyses were conducted.

**Results:**

A total of 4,545 participants were included, of whom 16.13% had OAB, with a mean SII of 5.75 ± 0.07. OAB risk increased with higher SII tertiles. In the fully adjusted model (Model 2), individuals in the highest SII tertile exhibited a 41% higher risk of OAB compared to those in the lowest tertile (OR: 1.41, 95% CI: 1.13–1.76, P = 0.004). Two-piece-wise regression analysis identified an SII breakpoint at 3.40, where a significant positive association was found for SII ≥ 3.40 (OR = 1.06, P < 0.0001), whereas no significant association was detected for SII < 3.40 (P = 0.06). Subgroup and interaction analyses revealed a consistent relationship between SII and OAB across different population strata, except for diabetes mellitus.

**Conclusion:**

SII, an easily accessible biomarker, was independently associated with an increased risk of OAB, highlighting its potential utility in diagnostic prediction.

## Introduction

Overactive bladder (OAB) is a prevalent urinary disorder characterized by urgency, increased daytime and/or nocturnal frequency, and, in some cases, incontinence, in the absence of a diagnosed urinary tract infection or other underlying conditions [1]. The incidence of OAB has risen significantly in recent years, likely due to escalating social stress and rapidly evolving lifestyles [2]. According to data from the five-country European Prospective Investigation into Cancer and Nutrition, which included over 19,000 adults, the overall OAB prevalence is estimated at 11.8%, with a higher occurrence in women than in men (12.8% vs. 10.8%) [3]. A study investigating OAB and its impact on occupational limitations reported that approximately 30% of American women experience OAB symptoms [4]. Additionally, OAB symptoms tend to be more pronounced in high-stress environments and significantly impair quality of life. The primary pathological mechanism in OAB is detrusor overactivity, whereas other forms of urethra–bladder dysfunction constitute a relatively small proportion of cases. Despite extensive research, the precise pathophysiology of OAB remains unclear, with no consensus on its etiology. Given the limited understanding of its underlying mechanisms, current long-term management strategies focus on symptom relief through lifestyle modifications, regular physical activity, pharmacological interventions (vaginal estrogen, anticholinergics, and β3 agonists), and invasive procedures (sacral neuromodulation, percutaneous tibial nerve stimulation, and surgical interventions) [5]. However, these treatments primarily alleviate symptoms rather than provide a definitive cure for OAB.

Emerging evidence suggests a strong association between OAB and various risk factors, including obesity, smoking, alcohol consumption, physical inactivity, diabetes mellitus (DM), depression, and lower socioeconomic status [6–8]. The immune system plays a critical role in the pathogenesis of numerous diseases, and accumulating research indicates that chronic, rather than acute, inflammation contributes to OAB development [9]. For instance, bladder biopsies from OAB patients have demonstrated the presence of inflammatory cells within the lamina propria and urothelium [10]. Additionally, urinary chemokines such as monocyte chemotactic protein-1 and inflammatory proteins are significantly elevated in OAB patients compared to healthy controls, reinforcing the notion that chronic inflammation is a key feature of OAB pathophysiology [11]. Emily Ma et al. further demonstrated the presence of an underlying systemic inflammatory component in OAB, characterized by an increased presence of proinflammatory markers and a reduced presence of anti-inflammatory markers [12]. Furthermore, platelets play a crucial role in modulating the inflammatory response [13]. Emerging studies suggest that combined peripheral lymphocyte, neutrophil, and platelet counts provide superior predictive value for inflammatory responses and can serve as key indicators of various diseases. The systemic immune-inflammatory index (SII) was initially introduced as a prognostic marker for conditions such as cancer, intracerebral hemorrhage, and coronary stenosis [14–16]. However, to date, no consensus has been reached regarding the influence of SII on OAB, and only limited studies have investigated its prognostic value in OAB patients.

The present study utilized data from the National Health and Nutrition Examination Survey (NHANES) to evaluate the correlation between SII and OAB risk among U.S. adults.

## Materials and methods

### Research participants and data collection

**Fig 1:**
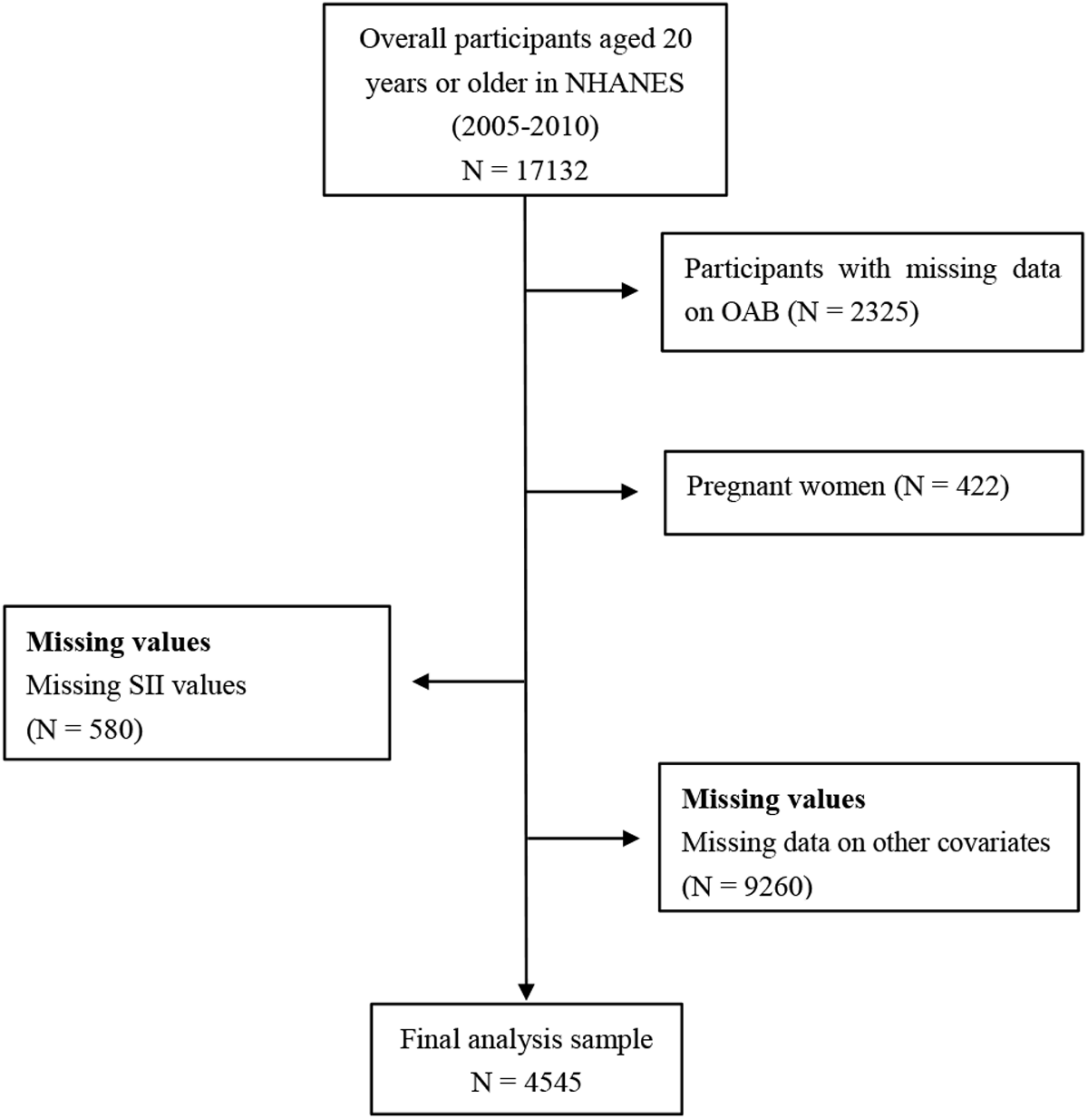
Representation of study design. **NHANES** National Health and Nutrition Examination Survey, **OAB** Overactive bladder, **SII** systemic immune-inflammatory index.

The analyzed data were obtained from the 2005–2010 NHANES, which utilized an extensive, multi-step, probability-clustering technique to acquire health and nutritional information from US adults and children. Participants were originally recruited into the NHANES database through its standardized selection process. For this investigation, subjects were identified within the NHANES population based on the availability of relevant data. Sampling included oversampling of participants from various racial and ethnic backgrounds, including non-Hispanic Black (NHB), non-Hispanic White (NHW), and Mexican American (MA). The initial dataset consisted of 17,132 individuals aged ≥20 years. For this investigation, exclusions were made for participants with missing OAB data (n = 2,325) and systemic immune-inflammation index (SII) data (n = 580). Pregnant women (n = 422) were also excluded to minimize confounding effects, as pregnancy-associated physiological changes, such as altered immune-inflammatory responses and bladder function, could influence both the systemic immune-inflammation index (SII) values and the assessment of OAB. Additionally, individuals with missing data on marital status, educational status, body mass index (BMI), smoking status, alcohol intake, family poverty-to-income ratio (PIR), hypertension, chronic kidney disease (CKD), fasting glucose (FG), and uric acid (UA) status (n = 9,260) were removed. After applying these inclusion and exclusion criteria, the final sample included 4,545 participants. The NHANES protocol received ethical approval from the National Center for Health Statistics (NCHS), and informed consent was obtained from all participants. The data utilized were publicly available from official NHANES sources (**Fig 1**). The NHANES protocol received ethical endorsement from the National Center for Health Statistics (NCHS), and obtained informed consent from all subjects [17–19]. The used information was adopted from a public website, and is also available from other sources [20].

### Exposure variables and outcomes

The SII hypothesis was first introduced by Hu et al. [21] to assess the prognostic performance of various diseases. SII is a composite inflammatory index derived from peripheral blood components, specifically neutrophils (N), lymphocytes (L), and platelets (P), and it was formulated as P × N/L (10^9/L) [21]. This index reflects the balance between inflammatory and immune responses, with higher values indicating a proinflammatory state. Due to the wide variability in SII values, a transformation was applied by dividing the original SII by 1000 for statistical analysis. In addition to the associations observed between SII and OAB, it is important to consider the potential for reverse causation in this relationship. Chronic inflammation, which is a hallmark of various conditions such as metabolic disorders and kidney disease, may not only be a consequence of OAB, but could also contribute to its development. This interaction suggests a bidirectional relationship, wherein the inflammatory response associated with OAB may elevate SII, creating a feedback loop that complicates the interpretation of causal direction. Further studies that assess the temporal dynamics of inflammation and OAB are warranted to better understand this complex relationship and mitigate potential reverse causation bias.

Based on the established definition of OAB, a diagnosis was made when a patient exhibited urge urinary incontinence and nocturia. The following three questions were adapted from the Kidney Conditions–Urology questionnaire in NHANES: [1] In the past 12 months, have you experienced leakage or loss of bladder control when feeling the urge to urinate but were unable to reach the bathroom in time? [2] How frequently has this occurred? [3] Over the past 30 days, how many times per night did you wake to urinate? To further refine OAB classification, the study utilized the *Criteria for Conversion of Symptom Frequencies Recorded Table* from NHANES and the Overactive Bladder Symptom Score (OABSS) from Shenhao Zhu et al. [22]. OAB symptom severity was quantified using a standardized scoring system, as outlined in Table 1. Finally, the total OABSS score for each participant was calculated by summing the nocturia and urge urinary incontinence scores. Individuals with a total score of ≥ 3 were classified as having OAB [23]. Given that NHANES does not provide a direct OABSS assessment, a modified scoring approach was developed using symptom frequency conversion tables from NHANES in accordance with validated OABSS criteria. The modification was based on established methodologies for symptom quantification in epidemiological datasets, where direct OABSS measurements are unavailable. The applied criteria align with previously validated OABSS scoring systems, ensuring consistency with existing literature.

**Table 1:** Criteria for conversion of symptom frequencies recorded in NHANES and OABSS scores.

According to NHANES score	According to OABSS score
**Urge urinary incontinence** **frequency**	**Urge urinary** **incontinence score**
Never	0
Less than once a month	1
A few times a month	1
A few times a week	2
Every day or night	3
**Nocturia frequency Nocturia** **score**	**Nocturia frequency** **Nocturia score**
0	0
1	1
2	2
3	3
4	3
5 or more	3

**Abbreviations:** NHANES, National Health and Nutrition Examination Survey; OABSS, Overactive Bladder Symptom Score.

### Biochemical parameters

Serum biochemical assessment was conducted, according to standard protocols [24,25] (33716004, 32319718), using blood from volunteer patients at a mobile examination center (MEC). Among the analyzed variables were UA, FG, creatinine and blood urea nitrogen (BUN).

### Other covariates of interest

Herein, we examined a range of possible OAB-related confounding factors identified according to epidemiological investigations. The analyzed socioeconomic parameters were marital status, education status, family poverty-to-income ratio (PIR), race, age, and gender; physical parameter was BMI; lifestyle parameters were alcohol intake and smoking habit; and comorbid parameters were DM, hypertension, hyperlipidemia, CKD and arteriosclerotic cardiovascular disease (ASCVD).

Sociodemographic variables (self-reports) were stratified as follows: age, < 45, 45–64, or ≥ 65 years; sex, male or female; race/ethnicity, NHB, NHW, MA, or others, encompassing a spectrum of racial backgrounds, as well as examination of marital status, categorizing cases as either never married, currently married, or living separately. The latter classification denoted those who were either divorced, widowed, or residing in distinct households; educational status, less than high school (HS), HS graduate, or beyond HS; BMI ≥ 25 kg/m^2 were considered overweight, including obese (based on World Health Organization (WHO) standards); PIR, < 1.00 represented below poverty threshold, PIR > 3.00 meant 3X above the poverty threshold.

Lifestyle variables (self-reports) were categorized as follows: alcohol intake, lifetime abstainers consumed <12 drinks in lifetime, former drinkers consumed ≥12 drinks in lifetime but quit the year prior to the study, current light drinkers consumed ≤3 drinks per week, and current heavy drinkers consumed >3 drinks per week, smoking habit (according to NCHS and CDC guidelines), never smokers or smoked <100 cigarettes in lifetime, consumed ≥100 cigarettes but quit before study, current smokers.

This study also investigated the comorbidities, including hypertension, hyperlipidemia, DM, CKD and ASCVD. The inclusion criteria for hypertension included medical diagnosis of hypertension, the anti-hypertensive drug usage, or a blood pressure reading ≥ 140/90 mmHg. Hyperlipidemia was described as high-density lipoprotein cholesterol (HDL-C) <40 mg/dL, low-density lipoprotein cholesterol (LDL-C) ≥160 mg/dL, triglycerides ≥200 mg/dL, and total cholesterol (TC) ≥240 mg/dL, or by a previous diagnosis obtained during the NHANES blood test. The participants were identified with type 2 DM based on specific criteria, including a medical diagnosis of diabetes, oral glucose tolerance ≥11.1 mmol/L, random glucose level ≥11.1 mmol/L, FG level ≥7.0 mmol/L, Hemoglobin A1c (HbA1c) ≥6.5%, or antidiabetic medication usage. The estimated glomerular filtration rate (eGFR) was determined via the Chronic Kidney Disease Epidemiology Collaboration (CKD-EPI) formula and CKD was described as eGFR < 60 ml/min/1.73 m^2, or a urine albumin-to-creatinine ratio (UACR) >30 mg/g

The examined participant comorbidities and their definitions are as follows: hypertension, required a medical diagnosis, anti-hypertensive drug usage, or systolic blood pressure (BP) ≥140 mmHg and/or diastolic blood pressure ≥90 mmHg; type 2 DM required a medical diagnosis, oral glucose tolerance ≥11.1 mmol/L, random glucose ≥11.1 mmol/L, fasting glucose ≥7.0 mmol/L, hemoglobin A1c (HbA1c) ≥6.5%, or antidiabetic drug usage; CKD, estimated glomerular filtration rate (eGFR) was computed based on the Chronic Kidney Disease Epidemiology Collaboration (CKD-EPI) formula, eGFR < 60 ml/min/1.73 m^2, or urine albumin-to-creatinine ratio (UACR) >30 mg/g [26]; hyperlipidemia was described as high-density lipoprotein cholesterol (HDL-C) <40 mg/dL, low-density lipoprotein cholesterol (LDL-C) ≥160 mg/dL, triglycerides ≥200 mg/dL, and total cholesterol (TC) ≥240 mg/dL, or by a previous diagnosis obtained during the NHANES blood test; ASCVD was determined via self-report with subject responding ‘Yes’ or ‘No’, including any one of coronary heart disease, angina, heart attack and stroke.

### Statistical analysis

All data analyses were conducted using R 4.2 software. To account for the complex, stratified, multistage clustering design of NHANES and mitigate the intentional oversampling of specific demographic groups, sampling weights were applied [27]. Survey-weighted continuous and categorical data were presented as mean ± standard error (SE) and as counts with percentages, respectively. The SII variable was transformed from a continuous measure into a categorical format. Consequently, multiple models were developed to evaluate the distinct effects of SII and OAB on patients’ outcomes. SII was analyzed both as a continuous variable and as a categorical variable based on tertiles. Inter-group differences, stratified by SII tertiles or OAB status, were assessed using the weighted Chi-square test for categorical data and the weighted Student’s *t*-test for continuous data. In this study, a robust analytical approach was implemented, incorporating weighted univariate and multivariate logistic regression analyses to elucidate the complex relationship between SII and OAB across various models. The crude model was unadjusted, while Model 1 was adjusted for age, sex, and race. Model 2 included a comprehensive set of covariates, such as age, sex, race, body mass index (BMI), marital status, education level, family poverty-income ratio (PIR), smoking status, alcohol consumption, hypertension, hyperlipidemia, chronic kidney disease (CKD), atherosclerotic cardiovascular disease (ASCVD), diabetes mellitus (DM), fasting glucose (FG), uric acid (UA), creatinine, and blood urea nitrogen (BUN). P-values were derived using logistic regression models. Multi-subgroup association heterogeneity was assessed through interaction analyses. Additionally, a restricted cubic spline (RCS) was employed to investigate the dose-response relationship between SII and OAB. In the presence of a nonlinear association, a two-piece linear regression model (segmented regression) was applied to fit individual intervals, quantifying threshold effects. A log-likelihood ratio test was conducted to distinguish between linear and nonlinear associations. Statistical significance was defined as *P* < 0.05.

## Results

### Subject profiles

A total of 4,545 subjects were included in the analysis. The average age was 46.95 ± 0.43 years, in which 67.84% were women and 32.16% were men. The mean SII was 5.75 ± 0.07. OAB was reported in 16.13% of participants, with prevalence increasing across SII tertiles (Tertile 1: 13.63%; Tertile 2: 15.12%; Tertile 3: 19.19%). Significant differences were found across SII tertiles for sex, ethnicity, marital status, smoking status, BMI, DM, hypertension, and CKD (*P* < 0.05). Compared with the lowest SII tertile, cases in higher SII tertiles were more likely to be female, of non-Hispanic White (NHW) ethnicity, living separately, and had a greater likelihood of BMI ≥ 25 kg/m², current smoking, DM, hypertension, and CKD. Conversely, cases with lower SII values were more frequently male, of non-Hispanic Black (NHB) or Mexican American (MA) ethnicity, married, with BMI < 25 kg/m², and former smokers (Table 2).

**Table 2:** Baseline characteristics of the study population.

Characteristics (weighted)		SII categories			
	Total (N = 4545)	T1	T2	T3	*P*-value
Fasting glucose (mmol/L)	5.80 ± 0.03	5.78 ± 0.06	5.75 ± 0.04	5.86 ± 0.06	0.25
Uric acid (µmol/L)	315.61 ± 1.46	317.28 ± 2.52	312.28 ± 2.20	317.32 ± 2.49	0.22
Creatinine (µmol/L)	75.71 ± 0.49	76.46 ± 0.52	74.86 ± 0.55	75.88 ± 0.92	0.07
Blood urea nitrogen (mmol/L)	4.52 ± 0.04	4.60 ± 0.05	4.47 ± 0.06	4.49 ± 0.05	0.16
Overactive bladder					< 0.001
No	3606(83.87)	1232(86.37)	1224(84.88)	1150(80.81)	
Yes	939(16.13)	282(13.63)	292(15.12)	365(19.19)	
Age (years, n (%))					0.12
<45	1891(46.51)	629(48.98)	645(45.92)	617(44.98)	
45-64	1531(36.01)	511(32.53)	510(38.11)	510(36.98)	
>=65	1123(17.48)	374(18.49)	361(15.98)	388(18.05)	
Sex, n (%)					< 0.0001
Male	1604(32.16)	617(38.53)	525(31.79)	462(27.13)	
Female	2941(67.84)	897(61.47)	991(68.21)	1053(72.87)	
Race, n (%)					< 0.0001
Mexican American	860(8.48)	304(10.34)	312(9.82)	244(6.96)	
Non-Hispanic Black	857(10.58)	378(15.77)	261(10.17)	218(8.20)	
Non-Hispanic White	2277(71.54)	645(69.06)	755(75.41)	877(80.68)	
Other races	380(4.28)	128(4.84)	132(4.60)	120(4.16)	
Marital status, n (%)					0.01
Married	2660(62.31)	921(63.95)	907(64.17)	832(59.19)	
Live separated	1151(20.78)	334(17.91)	387(20.57)	430(23.40)	
Never married	734(16.91)	259(18.13)	222(15.27)	253(17.41)	
Education level, n (%)					0.09
Less than high school	566(6.87)	208(7.57)	194(7.09)	164(6.07)	
High school	1920(38.57)	611(36.49)	637(37.14)	672(41.67)	
More than high school	2059(54.56)	695(55.94)	685(55.77)	679(52.26)	
Family PIR, n (%)					0.3
< 1	938(13.41)	317(14.06)	298(12.52)	323(13.69)	
1-3	2052(38.80)	684(39.01)	668(36.93)	700(40.38)	
> 3	1555(47.79)	513(46.93)	550(50.54)	492(45.93)	
BMI (kg/m^2, n (%))					0.02
<25	1346(33.10)	483(37.33)	425(32.01)	438(30.56)	
>=25	3199(66.90)	1031(62.67)	1091(67.99)	1077(69.44)	
Smoking status, n (%)					< 0.0001
Never	2444(52.61)	863(55.67)	851(56.66)	730(46.22)	
Former	1078(23.95)	370(25.77)	344(22.07)	364(24.19)	
Now	1023(23.44)	281(18.56)	321(21.27)	421(29.59)	
Alcohol usage, n (%)					0.16
Never	771(14.54)	275(15.92)	257(14.76)	239(13.17)	
Former	1200(22.11)	382(20.26)	392(21.07)	426(24.64)	
Moderate	1340(34.74)	431(33.59)	454(36.58)	455(34.00)	
Heavy	1234(28.61)	426(30.23)	413(27.59)	395(28.19)	
DM, n (%)					0.01
No	3590(84.50)	1196(85.87)	1213(86.10)	1181(81.85)	
Yes	955(15.50)	318(14.13)	303(13.90)	334(18.15)	
Hypertension, n (%)					0.003
No	2688(65.07)	915(67.98)	913(66.37)	860(61.39)	
Yes	1857(34.93)	599(32.02)	603(33.63)	655(38.61)	
Hyperlipidemia, n (%)					0.37
No	1182(28.05)	397(29.51)	394(28.07)	391(26.80)	
Yes	3363(71.95)	1117(70.49)	1122(71.93)	1124(73.20)	
CKD, n (%)					< 0.001
No	3687(86.00)	1256(87.90)	1262(87.54)	1169(82.95)	
Yes	858(14.00)	258(12.10)	254(12.46)	346(17.05)	
ASCVD, n (%)					0.67
No	4095(92.61)	1373(93.14)	1369(92.49)	1353(92.29)	
Yes	450(7.39)	141(6.86)	147(7.51)	162(7.71)	

Continuous data are shown as means and SE (standard error), while categorical data are presented as percentages. **Abbreviations: SII** systemic immune-inflammatory index, **PIR** Poverty income ratio, **BMI** Body mass index, **CKD** Chronic Kidney Disease**, DM** Diabetes mellitus, **ASCVD** arteriosclerotic cardiovascular disease.

Clinical and biochemical characteristics of subjects with and without OAB are presented in Supplementary Table 1. Cases with OAB exhibited elevated levels of UA, FG, BUN, and creatinine and were more likely to be female, older, of NHB ethnicity, living separately, with an education level below high school graduation, a family PIR ≤ 3, a BMI ≥ 25 kg/m², non-drinkers, and with comorbidities including DM, hypertension, hyperlipidemia, CKD, and ASCVD (all *P* < 0.05).

### Relationship between SII and OAB risk

As presented in Table 3, univariate analysis revealed that older age, female sex, BMI ≥ 25 kg/m², living separately, NHB ethnicity, and a history of DM, hypertension, hyperlipidemia, CKD, and ASCVD were all significantly associated with an increased likelihood of OAB (*P* < 0.05). Additionally, elevated SII, FG, UA, creatinine, and BUN levels were positively correlated with OAB. In contrast, never being married, higher educational attainment, a family PIR > 3, and moderate to heavy alcohol consumption were negatively associated with OAB (*P* < 0.01).

**Table 3:** Univariate logistic regression analysis of various variables.

Variables	OR(95% CI)	*P*-value
Age (versus <45, years)		
45-64	2.66(2.06,3.44)	<0.0001
>=65	6.07(5.09,7.24)	<0.0001
Sex (versus Male)		
Female	1.57(1.32,1.86)	<0.0001
Race (versus Mexican American)		
Non-Hispanic Black	1.64(1.22,2.22)	0.002
Non-Hispanic White	0.98(0.74,1.29)	0.86
Other races	1.29(0.76,2.18)	0.34
BMI (versus < 25, kg/m^2)		
>=25	1.68(1.36,2.07)	<0.0001
Marital status (versus Married)		
Live separated	1.90(1.62,2.24)	<0.0001
Never married	0.56(0.42,0.75)	<0.001
Education level (versus Less than high school)		
High school	0.58(0.46,0.73)	<0.0001
More than high school	0.35(0.27,0.46)	<0.0001
Family PIR (versus < 1)		
1-3	0.95(0.75,1.21)	0.69
> 3	0.48(0.34,0.66)	<0.0001
Smoking status (versus Never)		
Former	1.18(0.95,1.47)	0.13
Now	1.08(0.86,1.37)	0.48
Alcohol usage (versus Never)		
Former	1.06(0.84,1.35)	0.60
Moderate	0.67(0.50,0.89)	0.01
Heavy	0.37(0.28,0.51)	<0.0001
DM (versus No)		
Yes	3.12(2.45,3.96)	<0.0001
Hypertension (versus No)		
Yes	3.11(2.52,3.85)	<0.0001
Hyperlipidemia (versus No)		
Yes	2.04(1.66,2.51)	<0.0001
CKD (versus No)		
Yes	3.08(2.56,3.69)	<0.0001
ASCVD (versus No)		
Yes	3.78(2.78,5.13)	<0.0001
SII continuous	1.06(1.04,1.08)	<0.0001
Fasting glucose (mmol/L)	1.14(1.09,1.20)	<0.0001
Uric acid (µmol/L)	1.00(1.00,1.00)	0.02
Creatinine (µmol/L)	1.00(1.00,1.01)	0.01
Blood urea nitrogen (mmol/L)	1.17(1.11,1.23)	<0.0001

**Abbreviations: PIR,** poverty income ratio; **BMI,** body mass index; **SII,** systemic immune-inflammatory index; **CKD,** chronic kidney disease; **DM**, diabetes mellitus; **ASCVD**, arteriosclerotic cardiovascular disease; **OR,** odds ratio; **CI,** confidence interval.

Multivariate analysis demonstrated a significant association between exposure and outcome variables, which remained robust after adjusting for confounding factors (*P* < 0.05). The results of the multivariate regression analyses are summarized in Table 4. When analyzed as a continuous variable, SII levels were strongly associated with OAB risk across all models: crude model (odds ratio [OR] = 1.06, 95% confidence interval [CI]: 1.04–1.08, *P* < 0.0001), adjusted Model 1 (OR = 1.06, 95% CI: 1.04–1.09, *P* < 0.0001), and fully adjusted Model 2 (OR = 1.05, 95% CI: 1.03–1.07, *P* < 0.001). This significant positive association persisted when SII was categorized into tertiles. In fully adjusted Model 2, individuals in the highest SII tertile exhibited a 41% higher likelihood of developing OAB compared with those in the lowest tertile (OR = 1.41, 95% CI: 1.13–1.76, *P* = 0.004). However, no significant difference was observed between tertile 1 and tertile 2 (OR = 1.12; 95% CI: 0.88–1.42; *P* = 0.33). In this study, we observed a significant interaction between DM and SII in relation to the risk of OAB (P interaction = 0.01). While the overall trend suggests that higher SII levels are associated with increased OAB risk, this relationship appears to be attenuated in subjects with DM. Specifically, in the subgroup of individuals with DM, the odds ratio for the association between SII and OAB risk was close to 1 (OR = 0.99, 95% CI: 0.95–1.04, P = 0.77), indicating a lack of a strong association in this group. These findings suggest that DM may nullify or modify the influence of SII on OAB risk, underscoring the need to consider the role of metabolic conditions such as DM when evaluating the SII-OAB relationship. This interaction suggests that the impact of systemic immune-inflammatory responses, as measured by SII, on the development of OAB may be influenced by the presence of DM. Given the complex interplay between metabolic disturbances and inflammatory markers in DM, further investigations are warranted to understand the mechanistic pathways through which DM modulates the effect of SII on OAB. Clinically, this effect modification implies that management strategies for OAB might need to be tailored according to the presence of comorbid conditions such as DM.

**Table 4:** Relationship between SII and OAB, showed by weighted multivariate logistic regression.

	Crude model		Model 1		Model 2	
Overactive bladder	OR (95% CI)	*P*-value	OR (95% CI)	*P*-value	OR (95% CI)	*P*-value
SII continuous	1.06(1.04,1.08)	<0.0001	1.06(1.04,1.09)	<0.0001	1.05(1.03,1.07)	<0.001
SII categories						
T1	Ref		ref		ref	
T2	1.13(0.90,1.42)	0.29	1.17(0.94,1.47)	0.16	1.12(0.88,1.42)	0.33
T3	1.51(1.23,1.85)	<0.001	1.60(1.27,2.00)	<0.001	1.41(1.13,1.76)	0.004
*P* for trend		<0.001		<0.001		0.003

SII values were converted from continuous to categorical variables (tertiles). **OR** odds ratio, **CI** confidence interval, **Ref** reference. **Crude model:** SII (systemic immune-inflammatory index). **Model 1:** SII, age, sex, race. **Model 2:** SII, age, sex, race, BMI (Body mass index), marital status, education level, family PIR (Poverty income ratio), smoking status, alcohol usage, hypertension, hyperlipidemia, CKD (Chronic Kidney Disease), ASCVD (arteriosclerotic cardiovascular disease), DM (Diabetes mellitus), fasting glucose, uric acid.

**Fig 2:**
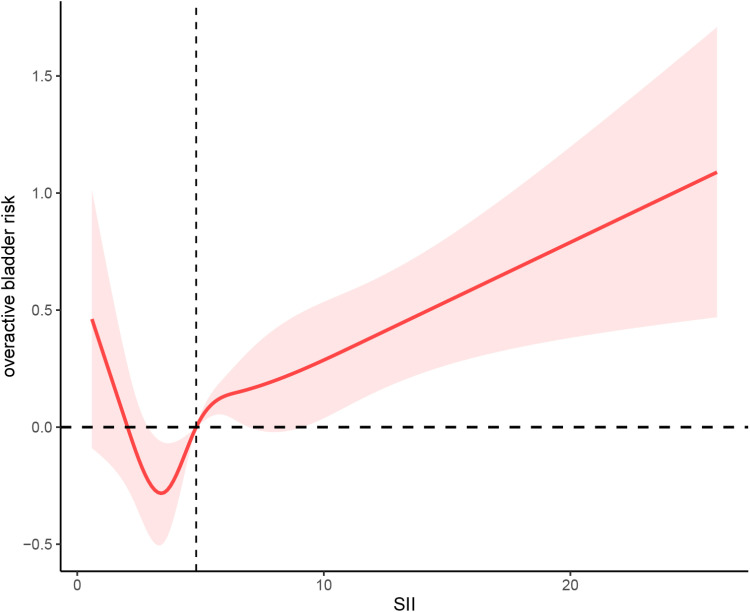
Restricted cubic spline analysis of the relationship between SII values and OAB risk.

Additionally, a marked nonlinear relationship was present between SII and OAB (**Fig 2**). Accordingly, we carried out a two-piece-wise regression model analysis and discovered that this model performed superior to the nonlinear model in explaining the crucial link between SII and OAB (*P* for log-likelihood ratio < 0.0001) (**Table 5**). The critical SII value was 3.40, with SII range between 0–3.40, SII rose by 1 unit, the OAB decreased by 0.25 (OR = 0.75, 95% CI: 0.55–1.01, *P* = 0.06); when the range of SII was ≥ 3.40, the relationship between SII and OAB was quite significant (OR = 1.06, 95% CI: 1.04–1.09, *P* < 0.0001).

**Table 5:** Results of binary logistic regression and piecewise linear regression model.

Outcome: overactive bladder	Adjusted OR (95%CI)	*P*–value
Fitting by binary logistic regression model	1.06(1.04, 1.08)	<0.0001
Fitting by the two–piecewise linear model		
Inflection point	3.40	
SII < 3.40	0.75(0.55, 1.01)	0.06
SII >= 3.40	1.06(1.04, 1.09)	<0.0001
Log–likelihood ratio	0.02	

No variables have been adjusted. **OR**, odds ratio; **CI**, confidence interval.

No variables have been adjusted.

### Stratified analysis

As presented in Table 6, subgroup analysis stratified by various confounding variables was performed. With the exception of DM (OR = 0.99, 95% CI: 0.95–1.04, *P* = 0.77), cases with higher SII consistently exhibited an increased risk of OAB compared with those with lower SII (all OR > 1), highlighting a strong association between continuous SII level and OAB risk.

**Table 6:** Subgroup analyses of the relationship between SII values and the prevalence of OAB.

Characteristics	OR(95% CI)	*P* for trend	*P* for interaction
Age (years)			0.28
<45	1.07(1.03,1.11)	0.001	
45-64	1.03(1.00,1.07)	0.05	
>=65	1.07(1.03,1.11)	<0.001	
Sex			0.44
Male	1.07(1.03,1.11)	<0.001	
Female	1.05(1.02,1.08)	<0.001	
Race			0.52
Mexican American	1.06(1.00,1.13)	0.07	
Non-Hispanic Black	1.06(1.01,1.12)	0.02	
Non-Hispanic White	1.06(1.03,1.09)	<0.0001	
Other races	1.14(1.04,1.24)	0.01	
Education level			0.15
Less than high school	1.11(1.05,1.18)	<0.001	
High school	1.04(1.01,1.07)	0.003	
More than high school	1.06(1.02,1.10)	0.002	
Marital status			0.73
Married	1.06(1.03,1.10)	<0.001	
Live separated	1.04(1.01,1.08)	0.03	
Never married	1.04(0.97,1.12)	0.31	
Family PIR			0.07
< 1	1.12(1.07,1.16)	<0.0001	
1-3	1.05(1.01,1.09)	0.01	
> 3	1.02(0.97,1.07)	0.38	
BMI (kg/m^2)			0.1
<25	1.09(1.04,1.13)	<0.001	
>=25	1.04(1.02,1.07)	0.001	
Smoking status			0.42
Never	1.07(1.04,1.11)	<0.0001	
Former	1.03(0.98,1.09)	0.27	
Now	1.06(1.01,1.10)	0.01	
Alcohol usage			0.59
Never	1.10(1.02,1.18)	0.01	
Former	1.07(1.03,1.11)	0.001	
Moderate	1.04(1.00,1.09)	0.07	
Heavy	1.06(1.02,1.09)	0.002	
DM			0.01
No	1.07(1.04,1.10)	<0.0001	
Yes	0.99(0.95,1.04)	0.77	
Hypertension			0.54
No	1.06(1.02,1.10)	0.004	
Yes	1.04(1.01,1.07)	0.01	
Hyperlipidemia			0.84
No	1.06(1.01,1.10)	0.02	
Yes	1.06(1.04,1.09)	<0.0001	
CKD			0.8
No	1.05(1.02,1.08)	0.001	
Yes	1.05(1.01,1.10)	0.02	
ASCVD			0.25
No	1.05(1.02,1.08)	<0.001	
Yes	1.09(1.03,1.15)	0.003	

**Abbreviations:** BMI (Body mass index), family PIR (Poverty income ratio), CKD (Chronic Kidney Disease), ASCVD (arteriosclerotic cardiovascular disease), DM (Diabetes mellitus). **OR** odds ratio, **CI** confidence interval, **Ref** reference.

Furthermore, potential effect modifications were evaluated by assessing interactions with age, sex, race, education level, BMI, marital status, smoking status, alcohol consumption, family PIR, DM, hypertension, hyperlipidemia, CKD, and ASCVD. Among these variables, only DM demonstrated a significant interaction effect (*P* for interaction = 0.01), while no other significant interactions were identified (*P* for interaction > 0.05). This suggests that the relationship between SII and OAB is largely independent of these potential effect modifiers (*P* for interaction > 0.05).

## Discussion

This observational analysis utilized standardized data from a large patient cohort in the USA. When SII exceeded 3.40, higher SII level was independently associated with an increased likelihood of OAB. Except for DM, subgroup and interaction analyses confirmed that this relationship remained unaffected by age, sex, race, education level, BMI, marital status, smoking status, alcohol consumption, family PIR, hypertension, hyperlipidemia, CKD, and ASCVD. These findings suggest that the combined assessment of peripheral neutrophil, lymphocyte, and platelet counts may facilitate the early identification of high-risk individuals predisposed to OAB, enabling timely interventions to mitigate disease development. While our findings demonstrate a significant association between SII and OAB, they do not establish a causal relationship. The presence of elevated SII values in OAB patients suggests a potential link, but further longitudinal and mechanistic studies are required to determine causation. Given the cross-sectional nature of NHANES data, our study cannot infer causality between SII and OAB. Future prospective studies and experimental research are needed to explore whether SII contributes to the development of OAB or is merely a coexisting phenomenon. It is important to note that correlation does not imply causation. The observed relationship between SII and OAB may be influenced by underlying confounders or shared inflammatory pathways, rather than a direct causal effect.

This investigation presents novel findings as the first to examine the correlation between SII and OAB. Chronic inflammation has been documented as a key factor associated with an increased likelihood of developing OAB [9]. Lymphocytes play a crucial role in the inflammatory response, with inflammation stimulating both their proliferation and activation, leading to an overall increase in lymphocyte count [28]. A reduction in peripheral lymphocyte count suggests immune cell dysfunction and potential apoptosis [29], which in turn contributes to an elevated SII. The present findings demonstrated a strong positive association between SII and OAB, particularly when the breakpoint exceeded 3.40, suggesting a potential protective role of lymphocytes against OAB. Neutrophils contribute to inflammation through the release of reactive oxygen species and antimicrobial peptides, forming neutrophil extracellular traps that may negatively impact bladder function [30]. This aligns with prior research indicating that elevated neutrophil-derived chemokines, such as GRO-α and MIP-1β, transfer from the urine to the bladder in OAB patients [11]. Additionally, platelets play a crucial role in regulating inflammation. Activated platelets initiate endogenous coagulation pathways, increasing susceptibility to various diseases, and further enhance the inflammatory state [31]. Platelets also interact with monocytes, neutrophils, and lymphocytes, modulating both innate and adaptive immune responses. Given that SII can be readily measured in routine clinical assessments, it provides an effective reflection of immune and inflammatory status. These findings highlight its potential utility as a convenient and reliable biomarker for assessing inflammation-driven conditions, including OAB.

SII is a relatively simple, novel, cost-effective, and easily accessible inflammatory parameter with high sensitivity but limited specificity. One of its key advantages is its ability to detect fluctuations in SII levels before clinical symptoms manifest, making it a potential early warning marker of pathological processes. Additionally, SII serves as an indicator of immune cell activation and systemic inflammation, with broad applicability in clinical medicine [14–16]. In this investigation, a strong association between SII and OAB was demonstrated, suggesting the potential utility of SII as a screening tool for OAB patients.

Despite the promising role of novel biomarkers such as SII, the predictive value of traditional risk factors (RFs) for OAB remains substantial. Detrusor overactivity is a primary contributor to OAB, and previous reports have indicated that smoking strongly exacerbates OAB pathology [32]. One proposed mechanism is that nicotine enhances sympathetic nervous system activity, thereby worsening irritative urinary symptoms [33]. Similarly, abdominal obesity, characterized by excessive body and visceral fat accumulation, increases intra-abdominal and bladder pressure, thereby accelerating OAB pathogenesis [34,35]. Importantly, moderate to heavy alcohol consumption was identified as a potential protective factor against OAB. Several studies [36] have reported a negative correlation between alcohol intake and OAB, whereas others [6] have indicated a direct association. Acute and chronic alcohol consumption may elevate circulating estrogen levels while reducing androgen levels [37], with these hormonal shifts contributing to urinary symptoms through multiple mechanisms [38]. Furthermore, animal model studies have demonstrated that ethanol reduces detrusor muscle and urethral contractility [39,40]. However, the precise mechanisms underlying these effects require further investigation.

Prior investigations established a strong correlation between OAB risk and DM. A well-known late-stage DM complication is peripheral neuropathy (PN). Diabetic PN stems from metabolic disarray of Schwann cells, which impairs axonal transfer, and damages nerve conduction and segmental demyelination. Hence, PN can cause detrusor overactivity, and is a potential RF for OAB [41]. Vascular and hypertensive RFs elevate ischemia, thereby introducing structural alterations within the bladder [42]. Moreover, prior reports suggested a strong inverse relation between educational status and OAB [6,43]. Authors revealed that individuals with a higher educational status pursued healthier options, however, those with lower educational status encounter poor diet, toxin exposure, and so on [44]. Here, the results observed in our study are almost consistent with the studies before. Therefore, although newly-discovered biomarkers may provide supplemental information, OAB risk stratification strongly relies upon classical RFs. The synergistic application of classical RFs and new inflammatory biomarkers like SII can potentially enhance overall OAB risk prediction accuracy. We recommend additional investigations to confirm this hypothesis.

The nonlinear relationship observed between the SII and OAB risk suggests that immune-inflammatory processes might not act in a simple linear fashion. At lower levels of SII, the immune response may be insufficient to contribute significantly to OAB, but as SII rises beyond a certain threshold, heightened inflammation and immune activation may begin to significantly impact bladder function and contribute to the development of OAB. The inflection point at an SII of 3.40 may correspond to a critical threshold where systemic inflammation and immune dysregulation reach a tipping point, thereby altering bladder function. Chronic inflammation is known to affect smooth muscle contractility and bladder sensitivity, potentially exacerbating symptoms of OAB in individuals with elevated SII. Given that the systemic immune-inflammatory index (SII) integrates neutrophil, lymphocyte, and platelet counts, it may serve as a proxy for the chronic low-grade inflammation observed in conditions such as diabetes, hypertension, and obesity, all of which are linked to OAB. It is plausible that elevated SII reflects an ongoing inflammatory milieu that predisposes individuals to bladder dysfunction, especially as inflammation progresses beyond a certain threshold. The significant association between higher SII and OAB risk, particularly among individuals with comorbid conditions such as diabetes, hypertension, and chronic kidney disease, aligns with previous studies suggesting that chronic inflammation may disrupt bladder control mechanisms. Further research is needed to elucidate the exact pathways through which systemic inflammation influences bladder smooth muscle activity and neurogenic control. The complex interplay between immune cells and neurogenic pathways may also explain the nonlinear association. Elevated SII may interact with neuroinflammatory processes, influencing bladder nerve function and increasing the likelihood of OAB development, particularly in the context of aging and comorbidities.

This research has several notable strengths. Firstly, data was acquired from NHANES, and all assessments involved recommended NHANES sample weight adjustments. Secondly, confounding factors were carefully adjusted to increase result reliability and to enable generalization to a wider population. Thirdly, owing to its relatively low cost, easy approach, and wide-ranging informative parameters, routine blood and blood biochemical evaluation unlocks great potential for OAB diagnosis and intervention. As a result, this screening method requires addition exploration and detailed analyses.

Nevertheless, this study had several limitations. Firstly, OAB status was determined through a questionnaire, which may have introduced recall, recording, and interviewer bias. Secondly, the NHANES database lacked detailed clinical information, such as prior medication use and OAB subtypes, both of which warrant further investigation. Additionally, blood samples were obtained from a single draw, whereas sequential testing would provide a more accurate representation of physiological conditions due to the dynamic nature of blood cell turnover. Thirdly, inherent limitations within the NHANES database may have led to unmeasured confounding factors influencing the results. Fourthly, since both exposure and outcome variables were assessed concurrently, the temporal relationship between SII and OAB remains unclear. Furthermore, the cross-sectional study design restricted the ability to establish a causal link between SII and OAB. We acknowledge the high exclusion rate of 73.5%, which may introduce selection bias. This exclusion rate resulted primarily from missing data, failure to meet eligibility criteria, and pre-existing medical conditions that could confound the study outcomes. To minimize potential bias, we performed sensitivity analyses to assess the robustness of our findings when different exclusion criteria were applied. These analyses did not substantially alter the overall results, suggesting that the impact of exclusion bias on the study’s conclusions is minimal. However, it is important to note that using publicly available repositories, such as NHANES may introduce certain biases. These include potential reporting biases, as many variables in the dataset rely on self-reported data, which could be influenced by participant recall bias or social desirability bias. Moreover, although NHANES employs a multi-stage, stratified sampling design, the dataset may still be subjected to selection bias, as it may not fully capture populations that are underrepresented or excluded from the survey (e.g., non-resident individuals, certain racial/ethnic subgroups). To mitigate these potential biases, we utilized sampling weights provided by NHANES to account for over- and under-sampling in different demographic groups, thereby improving the representativeness of the dataset. Furthermore, we conducted sensitivity analyses to assess the robustness of our findings and ensure the validity of our results across various subgroups and potential confounders. While some inherent limitations exist in the use of secondary data, we took comprehensive steps to minimize their impact on the conclusions drawn from this investigation. A key limitation of this study is the inability to calculate the prevalence of neurological disorders that may contribute to OAB, such as stroke, parkinsonism, spinal motor disorders, and peripheral neuropathy. Due to the design of the NHANES dataset, detailed clinical data or specific diagnostic codes necessary to precisely identify these conditions were not available. While this study concentrated on well-defined OAB-related comorbidities and socioeconomic factors, the potential role of neurological disorders in OAB pathophysiology warrants further investigation. Future research incorporating comprehensive neurological assessments will provide a clearer understanding of their contribution to OAB. This study relied on the CCI to assess comorbidity burden, as it is a well-validated and widely utilized measure in epidemiological research. While the frailty index could offer additional insights into the relationship between frailty and OAB by capturing broader physiological and functional decline, it was not included in the NHANES dataset used for this study. As a result, its integration was infeasible. Future research incorporating a frailty index may provide a more comprehensive understanding of the interplay between frailty and OAB. This study did not account for prior exposure to chemotherapy, radiotherapy, or immunotherapy, as NHANES does not provide comprehensive treatment history data for oncologic therapies. Given the potential impact of these treatments on systemic inflammatory markers, such as the SII, their omission represents a limitation. However, to minimize confounding effects, key covariates, such as chronic kidney disease, diabetes mellitus, and other inflammatory conditions were adjusted for in the multivariable models. Future research incorporating detailed treatment history data may help clarify the influence of prior oncologic therapies on SII values and their association with OAB.

## Conclusions

A significantly positive association was observed between elevated SII and increased OAB risk when SII exceeded 3.40. This suggests that SII may serve as a valuable marker for identifying high-risk individuals and guiding personalized treatment strategies. However, further large-scale prospective studies are required to confirm these findings.

## Supporting information

Supplementary Table 1 Baseline features of 4545 NHANES participants between 2005 and 2010. (DOCX)

## References

[1] Haylen BT, de Ridder D, Freeman RM, Swift SE, Berghmans B, Lee J, et al. An International Urogynecological Association (IUGA)/International Continence Society (ICS) joint report on the terminology for female pelvic floor dysfunction. Neurourol Urodyn. 2010;29(1):4-20. 19941278 10.1002/nau.20798

[2] Mingin GC, Heppner TJ, Tykocki NR, Erickson CS, Vizzard MA, Nelson MT. Social stress in mice induces urinary bladder overactivity and increases TRPV1 channel-dependent afferent nerve activity. Am J Physiol Regul Integr Comp Physiol. 2015;309(6):R629-38. doi: 10.1152/ajpregu.00013.2015 26224686 PMC4591369

[3] Irwin DE, Milsom I, Hunskaar S, Reilly K, Kopp Z, Herschorn S, et al. Population-based survey of urinary incontinence, overactive bladder, and other lower urinary tract symptoms in five countries: results of the EPIC study. Eur Urol. 2006;50(6):1306–14; discussion 1314-5. doi: 10.1016/j.eururo.2006.09.019 17049716

[4] Coyne KS, Sexton CC, Bell JA, Thompson CL, Dmochowski R, Bavendam T, et al. The prevalence of lower urinary tract symptoms (LUTS) and overactive bladder (OAB) by racial/ethnic group and age: results from OAB-POLL. Neurourol Urodyn. 2013;32(3):230–7. doi: 10.1002/nau.22295 22847394

[5] Beder D, Ashton P, Mishra V. Overactive bladder in women. BMJ. 2021;375:e063526. doi: 10.1136/bmj-2020-063526 34853012

[6] Pollard DR, Johnson WM, Lior H, Tyler SD, Rozee KR. Rapid and specific detection of verotoxin genes in Escherichia coli by the polymerase chain reaction. J Clin Microbiol. 1990;28(3):540–5. doi: 10.1128/jcm.28.3.540-545.1990 2182671 PMC269659

[7] Mckellar K, Bellin E, Schoenbaum E, Abraham N. Prevalence, Risk Factors, and Treatment for Overactive Bladder in a Racially Diverse Population. Urology. 2019;126:70–5. doi: 10.1016/j.urology.2018.12.021 30597170

[8] Hirayama A, Torimoto K, Mastusita C, Okamoto N, Morikawa M, Tanaka N, et al. Risk factors for new-onset overactive bladder in older subjects: results of the Fujiwara-kyo study. Urology. 2012;80(1):71–6. doi: 10.1016/j.urology.2012.04.019 22626577

[9] Antunes-Lopes T, Pinto R, Barros SC, Botelho F, Silva CM, Cruz CD, et al. Urinary neurotrophic factors in healthy individuals and patients with overactive bladder. J Urol. 2013;189(1):359–65. doi: 10.1016/j.juro.2012.08.187 23174241

[10] Apostolidis A, Jacques TS, Freeman A, Kalsi V, Popat R, Gonzales G, et al. Histological changes in the urothelium and suburothelium of human overactive bladder following intradetrusor injections of botulinum neurotoxin type A for the treatment of neurogenic or idiopathic detrusor overactivity. Eur Urol. 2008;53(6):1245–53. doi: 10.1016/j.eururo.2008.02.037 18343564

[11] Tyagi P, Barclay D, Zamora R, Yoshimura N, Peters K, Vodovotz Y, et al. Urine cytokines suggest an inflammatory response in the overactive bladder: a pilot study. Int Urol Nephrol. 2010;42(3):629–35. doi: 10.1007/s11255-009-9647-5 19784793

[12] Ma E, Vetter J, Bliss L, Lai HH, Mysorekar IU, Jain S. A multiplexed analysis approach identifies new association of inflammatory proteins in patients with overactive bladder. Am J Physiol Renal Physiol. 2016;311(1):F28-34. doi: 10.1152/ajprenal.00580.2015 27029431 PMC4967156

[13] Cetinkaya M, Buldu I, Kurt O, Inan R. Platelet-to-Lymphocyte Ratio: A New Factor for Predicting Systemic Inflammatory Response Syndrome after Percutaneous Nephrolithotomy. Urol J. 2017;14(5):4089–93. 28853103

[14] Liu J, Li S, Zhang S, Liu Y, Ma L, Zhu J, et al. Systemic immune-inflammation index, neutrophil-to-lymphocyte ratio, platelet-to-lymphocyte ratio can predict clinical outcomes in patients with metastatic non-small-cell lung cancer treated with nivolumab. J Clin Lab Anal. 2019;33(8):e22964. doi: 10.1002/jcla.22964 31282096 PMC6805305

[15] Trifan G, Testai FD. Systemic Immune-Inflammation (SII) index predicts poor outcome after spontaneous supratentorial intracerebral hemorrhage. J Stroke Cerebrovasc Dis. 2020;29(9):105057. doi: 10.1016/j.jstrokecerebrovasdis.2020.105057 32807462

[16] Liu Y, Ye T, Chen L, Jin T, Sheng Y, Wu G, et al. Systemic immune-inflammation index predicts the severity of coronary stenosis in patients with coronary heart disease. Coron Artery Dis. 2021;32(8):715–20. doi: 10.1097/MCA.0000000000001037 33826540

[17] Ciardullo S, Monti T, Perseghin G. Prevalence of Liver Steatosis and Fibrosis Detected by Transient Elastography in Adolescents in the 2017-2018 National Health and Nutrition Examination Survey. Clin Gastroenterol Hepatol. 2021;19(2):384-390.e1. doi: 10.1016/j.cgh.2020.06.048 32623006

[18] Vos MB, Abrams SH, Barlow SE, Caprio S, Daniels SR, Kohli R, et al. NASPGHAN Clinical Practice Guideline for the Diagnosis and Treatment of Nonalcoholic Fatty Liver Disease in Children: Recommendations from the Expert Committee on NAFLD (ECON) and the North American Society of Pediatric Gastroenterology, Hepatology and Nutrition (NASPGHAN). J Pediatr Gastroenterol Nutr. 2017;64(2):319–34. doi: 10.1097/MPG.0000000000001482 28107283 PMC5413933

[19] Friedewald WT, Levy RI, Fredrickson DS. Estimation of the Concentration of Low-Density Lipoprotein Cholesterol in Plasma, Without Use of the Preparative Ultracentrifuge. Clinical Chemistry. 1972;18(6):499–502. doi: 10.1093/clinchem/18.6.499 4337382

[20] Vilar-Gomez E, Nephew LD, Vuppalanchi R, Gawrieh S, Mladenovic A, Pike F, et al. High-quality diet, physical activity, and college education are associated with low risk of NAFLD among the US population. Hepatology. 2022;75(6):1491–506. doi: 10.1002/hep.32207 34668597

[21] Hu B, Yang X-R, Xu Y, Sun Y-F, Sun C, Guo W, et al. Systemic immune-inflammation index predicts prognosis of patients after curative resection for hepatocellular carcinoma. Clin Cancer Res. 2014;20(23):6212–22. doi: 10.1158/1078-0432.CCR-14-0442 25271081

[22] Zhu S, Wang Z, Tao Z, Wang S, Wang Z. Relationship Between Marijuana Use and Overactive Bladder (OAB): A Cross-Sectional Research of NHANES 2005 to 2018. Am J Med. 2023;136(1):72-8. 36150516 10.1016/j.amjmed.2022.08.031

[23] Xiao Y, Yin S, Wang J, Cui J, Yang Z, Wang J, et al. A positive association between the prevalence of circadian syndrome and overactive bladder in United States adults. Front Public Health. 2023;11:1137191. doi: 10.3389/fpubh.2023.1137191 37637821 PMC10449362

[24] Ciardullo S, Perseghin G. Statin use is associated with lower prevalence of advanced liver fibrosis in patients with type 2 diabetes. Metabolism: clinical and experimental. 2021;121:154752. 10.1016/j.metabol.2021.154752 33716004

[25] Zou B, Yeo YH, Nguyen VH, Cheung R, Ingelsson E, Nguyen MH. Prevalence, characteristics and mortality outcomes of obese, nonobese and lean NAFLD in the United States, 1999-2016. Journal of internal medicine. 2020;288(1):139–51. 32319718 10.1111/joim.13069

[26] KDIGO 2021 Clinical Practice Guideline for the Management of Glomerular Diseases. Kidney International. 2021;100(4s):S1-S276. 34556256 10.1016/j.kint.2021.05.021

[27] Akinbami LJ, Chen TC, Davy O, Ogden CL, Fink S, Clark J, et al. National Health and Nutrition Examination Survey, 2017-March 2020 Prepandemic File: Sample Design, Estimation, and Analytic Guidelines. Vital and health statistics Ser 1, Programs and collection procedures. 2022(190):1-36. 35593699

[28] Hotamisligil GS, Erbay E. Nutrient sensing and inflammation in metabolic diseases. Nat Rev Immunol. 2008;8(12):923–34. doi: 10.1038/nri2449 19029988 PMC2814543

[29] Kwon JH, Jang JW, Kim YW, Lee SW, Nam SW, Jaegal D, et al. The usefulness of C-reactive protein and neutrophil-to-lymphocyte ratio for predicting the outcome in hospitalized patients with liver cirrhosis. BMC Gastroenterol. 2015;15:146. doi: 10.1186/s12876-015-0378-z 26498833 PMC4619077

[30] Hermosilla C, Caro TM, Silva LMR, Ruiz A, Taubert A. The intriguing host innate immune response: novel anti-parasitic defence by neutrophil extracellular traps. Parasitology. 2014;141(11):1489–98. doi: 10.1017/S0031182014000316 24721985

[31] Mezger M, Nording H, Sauter R, Graf T, Heim C, von Bubnoff N, et al. Platelets and Immune Responses During Thromboinflammation. Frontiers in immunology. 2019;10:1731. 31402914 10.3389/fimmu.2019.01731 PMC6676797

[32] Dallosso HM, McGrother CW, Matthews RJ, Donaldson MMK, Leicestershire MRC Incontinence Study Group. The association of diet and other lifestyle factors with overactive bladder and stress incontinence: a longitudinal study in women. BJU Int. 2003;92(1):69–77. doi: 10.1046/j.1464-410x.2003.04271.x 12823386

[33] Platz EA, Rimm EB, Kawachi I, Colditz GA, Stampfer MJ, Willett WC, et al. Alcohol consumption, cigarette smoking, and risk of benign prostatic hyperplasia. Am J Epidemiol. 1999;149(2):106–15. doi: 10.1093/oxfordjournals.aje.a009775 9921955

[34] Al-Shaiji TF, Radomski SB. Relationship between Body Mass Index and Overactive Bladder in Women and Correlations with Urodynamic Evaluation. Int Neurourol J. 2012;16(3):126–31. doi: 10.5213/inj.2012.16.3.126 23094218 PMC3469831

[35] Zacche MM, Giarenis I, Thiagamoorthy G, Robinson D, Cardozo L. Is there an association between aspects of the metabolic syndrome and overactive bladder? A prospective cohort study in women with lower urinary tract symptoms. Eur J Obstet Gynecol Reprod Biol. 2017;217:1–5. doi: 10.1016/j.ejogrb.2017.08.002 28826038

[36] Dallosso HM, Matthews RJ, McGrother CW, Donaldson MMK, Shaw C, Leicestershire MRC Incontinence Study Group. The association of diet and other lifestyle factors with the onset of overactive bladder: a longitudinal study in men. Public Health Nutr. 2004;7(7):885–91. doi: 10.1079/phn2004627 15482614

[37] IARC Working Group on the Evaluation of Carcinogenic Risks to Humans. IARC monographs on the evaluation of carcinogenic risks to humans. Ingested nitrate and nitrite, and cyanobacterial peptide toxins. IARC Monogr Eval Carcinog Risks Hum. 2010;94:v–vii, 1–412. 21141240 PMC4781178

[38] Zhu L, Cheng X, Sun J, Lv S, Mei S, Chen X, et al. Association between Menopausal Symptoms and Overactive Bladder: A Cross-Sectional Questionnaire Survey in China. PLoS One. 2015;10(10):e0139599. doi: 10.1371/journal.pone.0139599 26448626 PMC4598107

[39] Ohmura M, Kondo A, Saito M. Effects of ethanol on responses of isolated rabbit urinary bladder and urethra. Int J Urol. 1997;4(3):295–9. doi: 10.1111/j.1442-2042.1997.tb00193.x 9255670

[40] Yokoi K, Ohmura M, Kondo A, Miyake K, Saito M. Effects of ethanol on in vivo cystometry and in vitro whole bladder contractility in the rat. J Urol. 1996;156(4):1489–91. doi: 10.1016/s0022-5347(01)65636-2 8808914

[41] Ikeda M, Nozawa K. Prevalence of overactive bladder and its related factors in Japanese patients with diabetes mellitus. Endocr J. 2015;62(9):847–54. doi: 10.1507/endocrj.EJ15-0237 26166691

[42] Torimoto K, Matsumoto Y, Gotoh D, Morizawa Y, Miyake M, Samma S, et al. Overactive bladder induces transient hypertension. BMC Res Notes. 2018;11(1):196. doi: 10.1186/s13104-018-3317-6 29580270 PMC5870500

[43] Teloken C, Caraver F, Weber FA, Teloken PE, Moraes JF, Sogari PR, et al. Overactive bladder: prevalence and implications in Brazil. Eur Urol. 2006;49(6):1087–92. doi: 10.1016/j.eururo.2006.01.026 16497431

[44] Jo JK, Lee S, Kim YT, Choi HY, Kim SA, Choi BY, et al. Analysis of the risk factors for overactive bladder on the basis of a survey in the community. Korean J Urol. 2012;53(8):541–6. doi: 10.4111/kju.2012.53.8.541 22949998 PMC3427838

