# Supplementary material for: Relationship between the systemic immune-inflammatory index and overactive bladder risk: A cross-sectional assessment involving United States Adults
Source: PLoS One. 2025 May 7;20(5):e0323052. doi: 10.1371/journal.pone.0323052 (PMC12057966; doi:10.1371/journal.pone.0323052)
Supplement: Supplementary Table 1 — (DOCX) [file pone.0323052.s001.docx]

| **Supplementary Table 1** **Baseline features of 4545 NHANES participants between 2005 and 2010** | | | | |
| --- | --- | --- | --- | --- |
| **Characteristics (weighted)** |  | **Overactive Bladder** | |  |
|  | **Total (N = 4545)** | **No (N = 3606)** | **Yes (N = 939)** | ***P*-value** |
| Fasting glucose (mmol/L) | 5.80±0.03 | 5.71±0.03 | 6.25±0.11 | < 0.0001 |
| Uric acid (µmol/L) | 315.61±1.46 | 313.83±1.46 | 324.83±4.48 | 0.02 |
| Creatinine (µmol/L) | 75.71±0.49 | 75.11±0.48 | 78.85±1.08 | 0.001 |
| Blood urea nitrogen (mmol/L) | 4.52±0.04 | 4.41±0.04 | 5.08±0.09 | < 0.0001 |
| SII, n (%) |  |  |  | < 0.001 |
| T1 | 1514(30.33) | 1232(31.23) | 282(25.62) |  |
| T2 | 1516(33.70) | 1224(34.11) | 292(31.59) |  |
| T3 | 1515(35.97) | 1150(34.66) | 365(42.79) |  |
| Age (years, n (%)) |  |  |  | < 0.0001 |
| <45 | 1891(46.51) | 1723(51.14) | 168(22.41) |  |
| 45-64 | 1531(36.01) | 1167(35.06) | 364(40.92) |  |
| >=65 | 1123(17.48) | 716(13.79) | 407(36.67) |  |
| Sex, n (%) |  |  |  | < 0.0001 |
| Male | 1604(32.16) | 1312(33.64) | 292(24.46) |  |
| Female | 2941(67.84) | 2294(66.36) | 647(75.54) |  |
| Race, n (%) |  |  |  | 0.003 |
| Mexican American | 860( 8.48) | 684(9.02) | 176(8.52) |  |
| Non-Hispanic Black | 857(10.58) | 629(10.22) | 228(15.85) |  |
| Non-Hispanic White | 2277(71.54) | 1845(76.39) | 432(70.33) |  |
| Other race | 380(4.28) | 300(4.36) | 80(5.30) |  |
| Marital status, n (%) |  |  |  | < 0.0001 |
| Married | 2660(62.31) | 2170(63.13) | 490(58.08) |  |
| Live separated | 1151(20.78) | 802(18.54) | 349(32.43) |  |
| Never married | 734(16.91) | 634(18.33) | 100( 9.49) |  |
| Education level, n (%) |  |  |  | < 0.0001 |
| Less than high school | 566(6.87) | 381(5.82) | 185(12.30) |  |
| High school | 1920(38.57) | 1488(37.26) | 432(45.41) |  |
| More than high school | 2059(54.56) | 1737(56.92) | 322(42.29) |  |
| Family PIR, n (%) |  |  |  | < 0.0001 |
| < 1 | 938(13.41) | 707(12.61) | 231(17.57) |  |
| 1-3 | 2052(38.80) | 1564(36.85) | 488(48.96) |  |
| > 3 | 1555(47.79) | 1335(50.54) | 220(33.48) |  |
| BMI (kg/m^2, n (%)) |  |  |  | < 0.0001 |
| <25 | 1346(33.10) | 1139(34.82) | 207(24.16) |  |
| >=25 | 3199(66.90) | 2467(65.18) | 732(75.84) |  |
| Smoking status, n (%) |  |  |  | 0.33 |
| Never | 2444(52.61) | 1961(53.11) | 483(50.00) |  |
| Former | 1078(23.95) | 827(23.53) | 251(26.14) |  |
| Now | 1023(23.44) | 818(23.36) | 205(23.86) |  |
| Alcohol usage, n (%) |  |  |  | < 0.0001 |
| Never | 771(14.54) | 570(13.61) | 201(19.39) |  |
| Former | 1200(22.11) | 884(20.42) | 316(30.92) |  |
| Moderate | 1340(34.74) | 1086(35.04) | 254(33.20) |  |
| Heavy | 1234(28.61) | 1066(30.94) | 168(16.49) |  |
| DM, n (%) |  |  |  | < 0.0001 |
| No | 3590(84.50) | 2985(87.46) | 605(69.11) |  |
| Yes | 955(15.50) | 621(12.54) | 334(30.89) |  |
| Hypertension, n (%) |  |  |  | < 0.0001 |
| No | 2688(65.07) | 2326(69.46) | 362(42.22) |  |
| Yes | 1857(34.93) | 1280(30.54) | 577(57.78) |  |
| Hyperlipidemia, n (%) |  |  |  | < 0.0001 |
| No | 1182(28.05) | 1017(30.09) | 165(17.42) |  |
| Yes | 3363(71.95) | 2589(69.91) | 774(82.58) |  |
| CKD, n (%) |  |  |  | < 0.0001 |
| No | 3687(86.00) | 3057(88.72) | 630(71.87) |  |
| Yes | 858(14.00) | 549(11.28) | 309(28.13) |  |
| ASCVD, n (%) |  |  |  | < 0.0001 |
| No | 4095(92.61) | 3341(94.61) | 754(82.27) |  |
| Yes | 450( 7.39) | 265( 5.39) | 185(17.73) |  |
| Continuous data are shown as mean and SE (standard error), while categorical data are presented as percentages. **Abbreviations: SII** systemic immune-inflammatory index, **PIR** Poverty income ratio, **BMI** Body mass index, **CKD** Chronic Kidney Disease**, DM** Diabetes mellitus, **ASCVD**, arteriosclerotic cardiovascular disease. | | | | |
